# Supplementary material for: Quasi-Solid-State SiO2 Electrolyte Prepared from Raw Fly Ash for Enhanced Solar Energy Conversion
Source: Materials (Basel). 2022 May 17;15(10):3576. doi: 10.3390/ma15103576 (PMC9143756; doi:10.3390/ma15103576)
Supplement: Supplementary file 1 [file materials-15-03576-s001.zip › materials-1677700-supplementary.pdf]

materials

Supplementary Materials

Quasi-Solid-State SiO_2 Electrolyte Prepared from Raw Fly Ash for Enhanced Solar Energy Conversion

Gyo Hun Choi [1,†], Jaehyeong Park [2,3,†], Sungjun Bae [2,*] and Jung Tae Park [1,*]

1 Department of Chemical Engineering, Konkuk University, 120 Neungdong-ro, Gwangjin-gu, Seoul 05029, Korea
2 Department of Civil and Environmental Engineering, Konkuk Univesity, 120 Neungdong-ro, Gwangjin-gu, Seoul 05029, Korea
3 Department of Civil and Environmental Engineering and Institute of Construction and Environmental Engineering, Seoul National University, Gwanak-ro, Gwanak-gu, Seoul 08826, Korea
* Correspondence: bsj1003@konkuk.ac.kr (S.B.); jtpark25@konkuk.ac.kr (J.T.P.); Tel.: +82-2-450-3904 (S.B.); +82-2-450-3538 (J.T.P.)
† These authors contributed equally to this work.

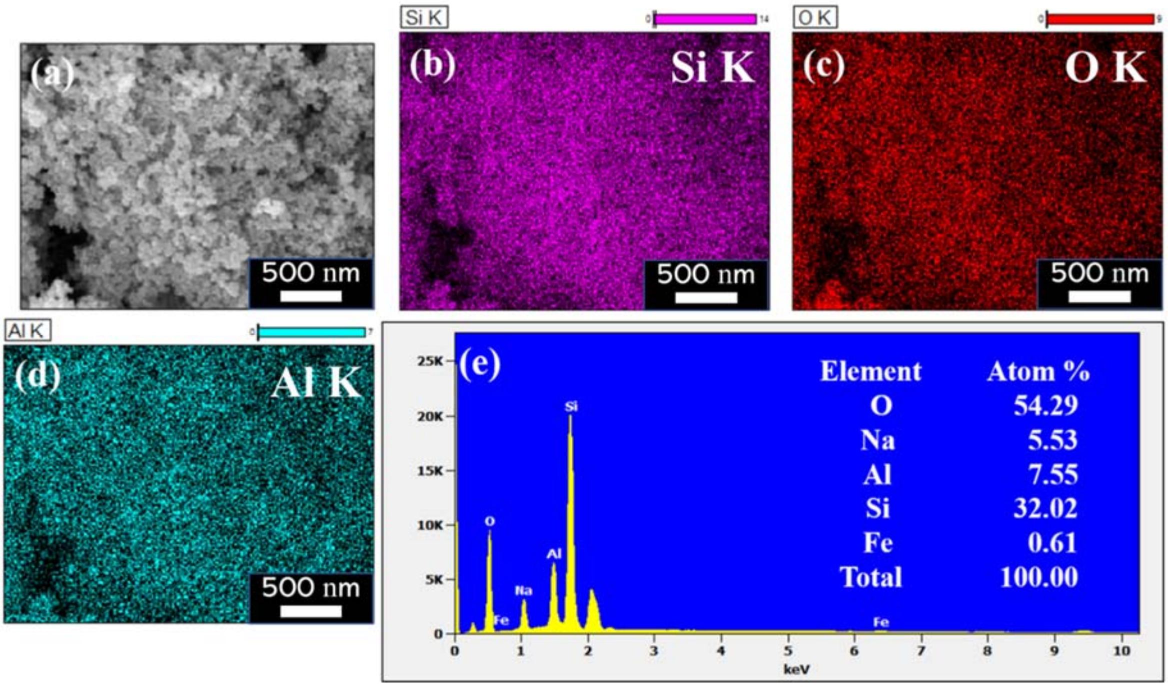

Figure S1. EDS analysis of the prepared FA_SiO_2.

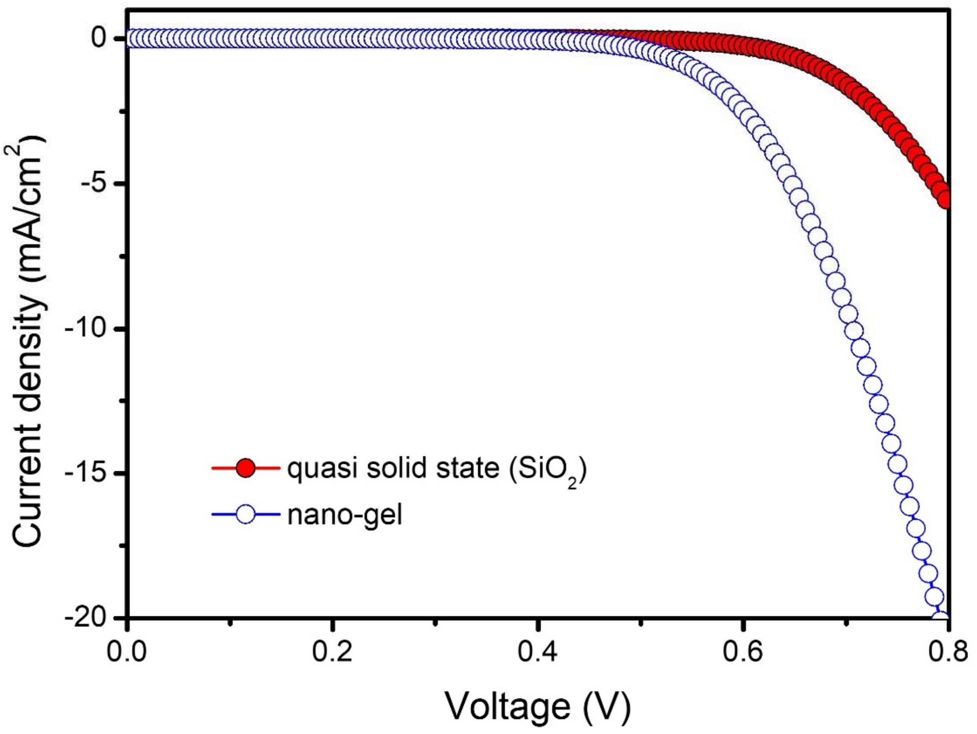

Figure S2. Photocurrent density-photovoltaic curves in the dark for DSSCs based on nano-gel and quasi solid state (SiO_2) electrolytes.

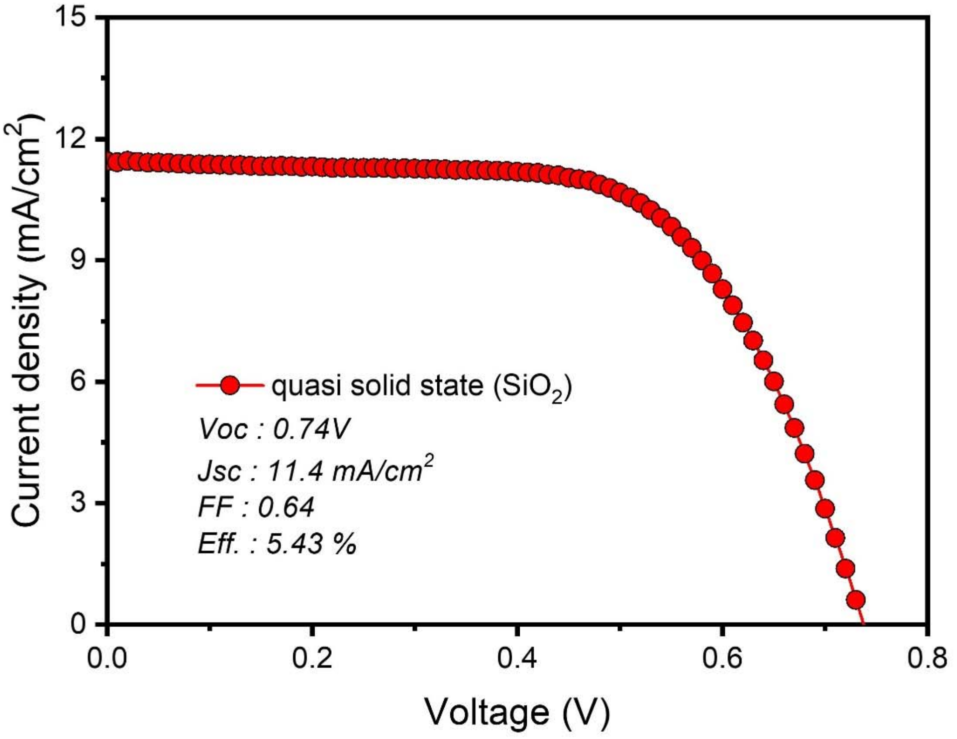

Figure S3. Photocurrent density-photovoltaic curves of DSSCs based quasi solid state (SiO_2) electrolytes at 100 mW cm^{-2} from different batches. Different cells were made for reliability test.

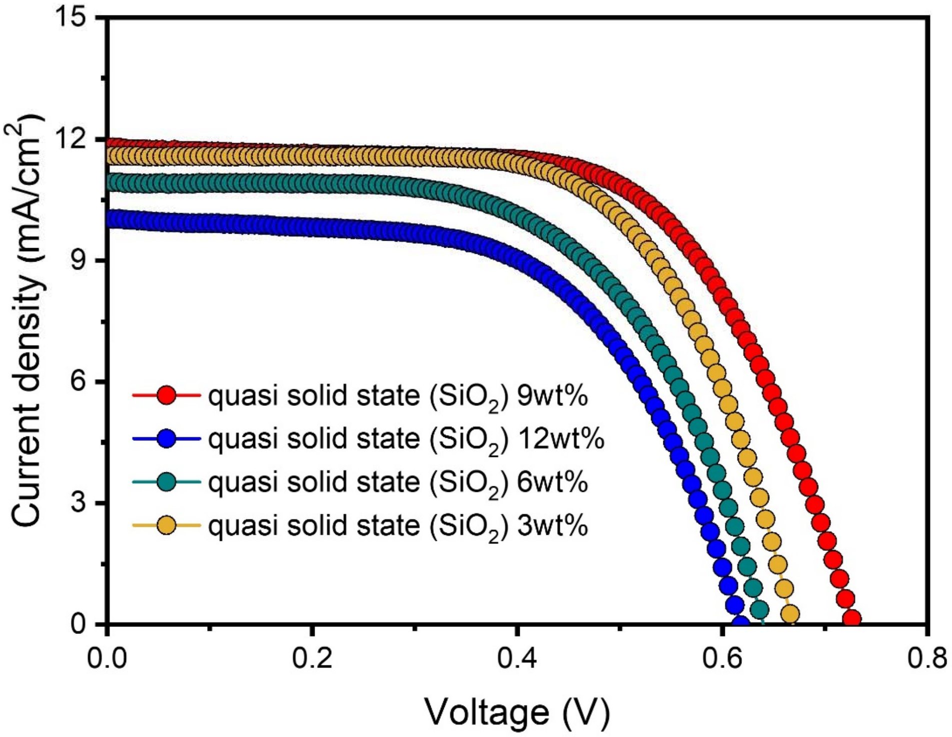

Figure S4. Photocurrent density-photovoltaic curves of DSSCs based on quasi solid state (SiO_2) electrolytes with different FA_SiO_2 content at 100 mW cm^{-2}.

Table S1. DSSCs electrolyte formulations.

Electrolyte	FA_SiO_2	PEG	LiI	MPII	I_2	Acetonitrile
Nano-gel	-	1 g	0.15 g	0.15 g	0.03 g	10 ml
Quasi solid state (SiO_2)	0.09 g	1 ml	0.15g	0.15 g	0.03 g	10 ml

Table S2. Comparison of photovoltaic parameters of DSSCs fabricated with quasi solid state electrolytes reported in the literature.

Quasi solid state electrolyte	*Voc* (V)	*Jsc* (mA/cm^2)	*FF*	η (%)	Reference
FA_SiO_2	0.73	12.1	0.62	5.5	This work
ZrO_2-C/PGEs	0.66	14.0	0.61	5.6	[41]
PHS	0.56	8.69	0.70	3.4	[42]
PGMA/PIN	0.84	20.37	0.47	8.0	[43]
P3/ES	0.51	4.8	0.62	1.5	[44]
P-WO_3	0.71	14.6	0.61	6.3	[45]

Table S3. Photovoltaic properties of DSSCs based on quasi solid state (SiO_2) electrolytes with different FA_SiO_2 contents under 1 sun illumination. (AM 1.5 G, 100 mW/cm^2).

	Voc **(V)**	***Jsc*** **(mA/cm^2)**	***FF***	***η (%)***
Quasi solid state (SiO_2) 3wt%	0.67	11.6	0.65	5.0
Quasi solid state (SiO_2) 6wt%	0.64	10.9	0.60	4.2
Quasi solid state (SiO_2) 9wt%	0.73	12.1	0.62	5.5
Quasi solid state (SiO_2) 12wt%	0.62	10.0	0.60	3.7
